# Advances in graft-versus-host disease prevention strategies: latest updates from the 2022 ASH annual meeting

**DOI:** 10.1186/s40164-023-00425-y

**Published:** 2023-07-07

**Authors:** Yiyin Chen, Yang Xu, Depei Wu

**Affiliations:** National Clinical Research Center for Hematologic Diseases, Jiangsu Institute of Hematology, The First Affiliated Hospital of Soochow University, Institute of Blood and Marrow Transplantation, Collaborative Innovation Center of Hematology, Soochow University, Suzhou, Jiangsu China

**Keywords:** Hematopoietic stem cell transplantation, Graft-versus-host disease, Prophylaxis

## Abstract

This review summarizes the significant advancements in prophylaxis against graft-versus-host disease (GvHD) presented at the 2022 ASH Annual Meeting. The use of innovative agents and regimens, along with the conventional prophylactic approach of combining post-transplant cyclophosphamide and anti-thymocyte globulin, were discussed. The innovative agents and regimens highlighted in this review include abatacept, the first FDA-approved drug for acute GvHD prophylaxis; RGI-2001, which promotes the expansion of regulatory T cells; and cell therapies such as Orca-T and Orca-Q. These advancements provide promising strategies and options for GvHD prevention, offering hope for improved post-transplant patient outcomes in terms of survival rates.

## To the editor

Hematopoietic stem cell transplantation (HSCT) can lead to a severe multisystem disorder known as graft-versus-host disease (GvHD), resulting in considerable morbidity and non-relapse mortality (NRM). We reviewed several latest reports on GvHD prophylaxis from the 2022 ASH Annual Meeting (ASH2022).

## Novel agents and regimens

Abatacept (ABA), a CD28:CD80/86 co-stimulation blockade, has shown potential in controlling early T cell allo-proliferative escape, a major contributor to breakthrough acute GvHD (aGvHD) following calcineurin inhibitor/methotrexate (CNI/MTX) prophylaxis [1]. ABA + CNI/MTX administration in recipients of 7/8 mismatched unrelated donor (MMUD) grafts, known to carry a higher risk of GvHD-related mortality, demonstrated comparable overall survival (OS) and recurrence-free survival (RFS) rates to those of 8/8 matched unrelated donor (MUD) recipients, thereby improving the risk/benefit ratio for MMUD HSCT [2]. A real-world study further confirmed the finding in adult and pediatric patients [3]. Moreover, ABA/Tacrolimus +- post-transplant cyclophosphamide (PTCy) demonstrates feasibility and promising outcomes with low GvHD rates in matched or mismatched donor peripheral blood stem cell transplantation (PBSCT) [4].

The augmentation of regulatory T cells (Tregs) has gained significant attention as an effective strategy for preventing GvHD while preserving the graft-versus-leukemia (GvL) effect. RGI-2001, a liposomal glycolipid, expands Tregs by activating invariant natural killer cells through CD1d receptor on antigen-presenting cells, thereby modulating GvHD pathogenesis. RGI-2001 administered intravenously with tacrolimus/MTX was effective in preventing aGvHD in individuals receiving grafts from peripheral blood stem cell (PBSC) and bone marrow sources, with a favorable safety profile. Promising results justify the planning of a phase 3 clinical trial to assess the efficacy of the regimen [5]. Orca-T is an innovative immunotherapy with purified donor regulatory T cells to target alloreactive immune responses. Graft composition optimization in HLA-matched PBSCT has shown robust GvL and graft-versus-infection effects, while also reducing the incidence of GvHD and NRM [6]. In the context of haplo-PBSCT, another cellular therapy, Orca-Q, has emerged as a potential prevention option. Administered with single-agent tacrolimus, Orca-Q shows low incidence and severity of GvHD, improved GvHD-free and relapse-free survival (GRFS) rates, and demonstrates clinical superiority even without the use of PTCy. However, the broad adoption of these cell therapies is hindered by the logistical challenge of timely vein-to-vein administration within a restricted 72-hour window, confining their implementation to the specialized institution [7] (Table 1).

**Table 1:** Applications of novel agents and regimens

Agent	Regimen	Mechanism	Study	Patients	Donors	GvHD
Abatacept	ABA/Tacrolimus +- PTCy	CD28:CD80/86 co-stimulation blockade	Single-center phase 1b-2 study (NCT04503616)	AML (n = 12) B-NHL (n = 9) ALL/CLL (n = 10) MDS (n = 5) Other (n = 15)	Matched and mismatched Related and unrelated	13.7% Grade II-IV aGvHD 9.8% Grade II-IV cGvHD
RGI-2001	100 ug/kg intravenous infusion weekly x 6 doses, starting on the day of transplant (Days 0, 7, 14, 21, 28, 35) + CNI + MTX/MMF	Cytokine-dependent Treg proliferation	Multi-center phase 2b study (NCT04014790)	AML (n = 27) ALL (n = 11) MDS (n = 7)	Matched related (n = 15) Matched unrelated (n = 28) Mismatched unrelated (n = 6)	20.4% Grade II-IV aGvHD 14.3% moderate cGvHD
Orca-T	Orca-T + tacrolimus (n = 124) /sirolimus (n = 3)	Polyclonal donor Tregs to control alloreactive immune responses	Single-center phase 2 study (NCT01660607) Multi-center Phase 1b study (NCT04013685)	AML (n = 62) ALL (n = 42) MPAL (n = 4) CML with prior blast crisis (n = 4) High risk MDS (n = 15)	Matched related (n = 66) Matched unrelated (n = 61)	5% Grade III-IV aGvHD 6% moderate/severe cGvHD
Orca-Q	Orca-Q + tacrolimus (PTCy was not permitted)	Deplete certain T-cell subsets (unreported) from the graft	Multi-center phase 1 study (NCT03802695)	AML (n = 11) ALL (n = 8) CML with blast crisis (n = 2)	Haplo (defined as ≥ 4/8 but < 7/8 matched)	14% Grade II-IV aGvHD 6.3% mild cGvHD

*ABA abatacept, PTCy post-transplant cyclophosphamide, CNI calcineurin inhibitor, MTX methotrexate, MMF mycophenolate mofetil, AML acute myeloid leukemia, B-NHL B-cell non-Hodgkin lymphoma, ALL acute lymphoblastic leukemia, CLL chronic lymphocytic leukemia, MDS myelodysplastic syndromes, MPAL mixed phenotype acute leukemia, CML chronic myeloid leukemia*

## PTCy and ATG combination prophylaxis

Combining anti-thymocyte globulin (ATG) and PTCy as prophylaxis against GvHD is a promising strategy, surpassing the limitations of individual administration. ATG-based regimens pose viral infection risks, while PTCy-based prophylaxis is less effective with PBSC grafts. A retrospective study [8] showed that incorporating ATG to the Clo-Baltimore (CloB) regimen with PTCy enhanced GRFS. Another study [9] indicated that a low-dose ATG (2.5 mg/kg)/PTCy (50 mg/kg) regimen holds promise for preventing GvHD following haplo-PBSCT, with a cumulative incidence of aGvHD observed at 30.7%.

Salas et al. conducted trials investigating the use of reduced-dose ATG in various transplantation settings. In Abstract 2127 [10], a comparison was made between two doses of ATG, specifically 4.5 mg/kg and 2 mg/kg, in the context of PBSCT from 10/10 MUD. The higher dose showed a decreased incidence of moderate/severe cGvHD, but was associated with an increased occurrence of infectious complications. Conversely, recipients of the reduced-dose ATG exhibited a trend towards improved 1-year OS. Another study focusing on haplo-PBSCT [11] also reported an increased risk of grade III-IV aGvHD with the administration of the reduced-dose ATG. These studies suggest that the use of low-dose ATG involves certain compromises in prevention efficacy.

Low-dose PTCy has been investigated in elderly patients and those with cardiac comorbidities undergoing haplo-PBSCT [12]. The study suggested that reducing PTCy to 70 mg/kg combined with low-dose ATG is a secure and efficacious strategy, resulting in higher GRFS. Furthermore, the low-dose PTCy led to accelerated immune reconstitution, decreased cumulative incidence of bacteremia, BK-virus associated hemorrhagic cystitis, and cardiac complications, while the risk of aGvHD and cGvHD remained unaffected (Table 2).

**Table 2:** Clinical outcomes of PTCy and ATG combination prophylaxis

Regimen	Patients	Donors	Survival	GvHD	Reference
CloB + ATG (n = 30)	AML (n = 32) MDS (n = 18) MPN (n = 5) MDS/MPN (n = 10)	Haploidentical (n = 54) Matched (n = 12)	70% 18-month GRFS	23.3% aGvHD 0% extensive cGvHD	[8]
CloB (n = 36)			43% 18-month GRFS	50% aGvHD 11% extensive cGvHD	
PTCy + ATG	AML(n = 113) ALL (n = 49) MPAL (n = 4) MDS (n = 18) CML (n = 10) NHL (n = 30) MM (n = 1)	Haploidentical	48% 2-year GRFS 60.9% 2-year OS 54.8% 2-year DFS 16.6% 2-year NRM	30.7% aGvHD 31.2% cGvHD	[9]
PTCy + ATG (2 mg/kg) + CsA (n = 233)	Unknown (n = 444)	Matched unrelated (10/10)	56.2% 1-year GRFS 78.3% 1-year OS 69.2% 1-year RFS 14.7% 1-year NRM	23.3% Grade II-IV aGvHD 8% Grade III-IV aGvHD 14.1% moderate/severe cGvHD	[10]
PTCy + ATG (4.5 mg/kg) + CsA (n = 127)			53.5% 1-year GRFS 72.4% 1-year OS 66.1% 1-year RFS 17.3% 1-year NRM	16.5% Grade II-IV aGvHD 4.9% Grade III-IV aGvHD 5.4% moderate/severe cGvHD	
PTCy + ATG	AML (n = 79) Unknown (n = 78)	Haploidentical	35.7% 2-year GRFS 49.4% 2-year OS 44.6% 2-year RFS 36.6% 2-year NRM	26.3% Grade II-IV aGvHD 9.5% Grade III-IV aGvHD 19.9% moderate/severe cGvHD	[11]
PTCy (70 mg/kg) + ATG (n = 33)	(1) age ≥ 65 years, or patients of any age with a history of cardiac events, (2) AML (n = 37), MDS (n = 11), lymphoma (n = 8), MPN (n = 2)	Haploidentical	60% 2-year GRFS 68% 2-year OS 65% 2-year PFS 18% 2-year NRM	18% Grade II-IV aGvHD 0% Grade III-IV aGvHD 27% cGvHD	[12]
PTCy (100 mg/kg) + ATG (n = 25)			33% 2-year GRFS 52% 2-year OS 46% 2-year PFS 33% 2-year NRM	17% Grade II-IV aGvHD 5% Grade III-IV aGvHD 29% cGvHD	

*AML acute myeloid leukemia, MDS myelodysplastic syndromes, MPN myeloproliferative neoplasms, ALL acute lymphoblastic leukemia, MPAL mixed phenotype acute leukemia, CML chronic myeloid leukemia, NHL non-Hodgkin’s lymphoma, MM multiple myeloma, ATG anti-thymocyte globulin, PTCy post-transplant cyclophosphamide, CsA cyclosporine, GRFS GvHD-free and relapse-free survival, OS overall survival, DFS disease-free survival, RFS recurrence-free survival, PFS progression-free survival, NRM non-relapse mortality, aGvHD acute graft-versus-host disease, cGvHD chronic graft-versus-host disease*

The emergence of novel agents and regimens has expanded the donor pool and allowed the use of different graft sources, including mismatched donors and PBSC, to address patient needs while minimizing the risk of GvHD. For PTCy and ATG combination prophylaxis, future endeavors could focus on investigating lower-dose combination of these agents to achieve the dual goals of minimizing adverse events and maintaining prophylactic efficacy.

## Data Availability

Not applicable.
